# Dynamic nomogram for predicting the overall survival and cancer-specific survival of patients with gastrointestinal neuroendocrine tumor: a SEER-based retrospective cohort study and external validation

**DOI:** 10.3389/fonc.2025.1594591

**Published:** 2025-06-04

**Authors:** Yipu Wang, Gongning Wang, Chao Song, Wenqian Ma, Xiuli Zheng, Shuo Guo, Qi Wang, Lan Zhang, Limian Er

**Affiliations:** ^1^ Department of Endoscopy, The Fourth Hospital of Hebei Medical University, Shijiazhuang, Hebei, China; ^2^ The Third Department of Surgery, The Fourth Hospital of Hebei Medical University, Shijiazhuang, Hebei, China; ^3^ Department of Gastroenterology, The Fourth Hospital of Hebei Medical University, Shijiazhuang, Hebei, China

**Keywords:** gastrointestinal neuroendocrine tumor (GI-net), surveillance epidemiology and end results (SEER) database, nomogram, overall survival (OS), cancer-specific survival (CSS)

## Abstract

**Background:**

Gastrointestinal neuroendocrine tumor (GI-net) is a rare heterogeneous tumor, and there is a lack of models to predict its prognosis. Our study aims to develop and validate two new nomograms to predict the overall survival (OS) and cancer-specific survival (CSS) of GI-net patients and investigate their application value.

**Methods:**

SEER*Stat 8.4.4 software was used to download clinicopathological information of GI-net patients between 2010 and 2015 from the Surveillance, Epidemiology, and End Results (SEER) database. These patients were randomly divided into a training group (n=3007) and an internal-validation group (n=1289) at a 7:3 ratio. Patients from the Fourth Hospital of Hebei Medical University were enrolled in this study to form the external-validation group (n=86). Univariate and multivariate Cox analyses were performed to explore the independent prognostic factors and establish two nomograms. The concordance index (C-index), area under the time-dependent receiver operating characteristic curve (AUC), calibration curve, and decision curve analysis (DCA) were used to evaluate the nomograms. X-tile was used to divide GI-net patients into high-, medium-, and low-risk groups. Kaplan–Meier (KM) curves and log-rank tests were used to compare survival differences among the three groups.

**Results:**

Seven variables (age, site, size, grade, M stage, surgery, and chemotherapy) were selected to establish the nomogram for OS, and 6 variables (age, size, grade, M stage, surgery, and chemotherapy) were selected for CSS. The C indices (0.785, 0.813, and 0.936 in the training, internal-validation, and external-validation groups for OS; 0.888, 0.893, and 0.930 for CSS, respectively) and AUCs (≥0.7) indicated that the nomograms had satisfactory discriminative ability. Calibration curve analysis and DCA revealed that the nomogram had a satisfactory ability to predict OS and CSS. KM curves indicated that each of the two nomograms clearly differentiated the high-, medium-, and low-risk groups. In addition, two online risk calculators were developed to predict the OS and CSS of these patients visually.

**Conclusions:**

Our nomograms may play an important role in predicting 3- and 5-year OS and CSS for GI-net patients. Risk stratification systems and online risk calculators can be utilized in clinical practice to help doctors create personalized treatment plans.

## 1 Background

Neuroendocrine neoplasms (NENs) are relatively rare and heterogeneous types of tumors (1–3), accounting for approximately 2% of all malignant tumors (1). These cells exhibit neuroendocrine differentiation and can secrete various hormones (2). The World Health Organization (WHO) classified NENs in 2022 into well-differentiated neuroendocrine tumors (NETs) and poorly differentiated neuroendocrine carcinomas (NECs) (4). Many studies have shown that NETs and NECs are significantly different in terms of their definitions, clinical pathological characteristics, treatments, and prognoses; thus, NETs should be studied independently.

GI-nets are the most common type of NETs (5–7). They typically have a hidden onset and slow growth, and their occurrence is associated with mutations in the MEN1 gene, the mTOR pathway, or others (8). In recent years, increased health awareness among the population and advancements in diagnostic technologies, particularly computed tomography and endoscopy, have resulted in a rising overall incidence of gastrointestinal neuroendocrine tumors (6, 9, 10). A population-based study revealed that the age-adjusted annual incidence of NETs increased from 1.09 per 100,000 people in 1973 to 6.98 per 100,000 people in 2012, a growth of 6.4-fold (6). The greatest increases in incidence have been observed in the stomach (15 times) and the rectum (9 times) for NETs (6). Gastrointestinal NETs are the second most common type of digestive system cancer (11, 12). GI-nets show two main types of clinical manifestations (4, 11). The first type includes local symptoms, such as obstructions, bleeding, and perforations, caused by tumor growth. The second type involves systemic symptoms, like carcinoid syndrome and Zollinger-Ellison syndrome, resulting from hormones or bioactive substances released by the tumor. The diagnosis relies on imaging tests and histological evaluations, with endoscopy and biopsy as the gold standard methods. In addition, biomarkers play an important role in the diagnosis and prognosis of GI-nets, showing significant future potential. Chromogranin A (CgA) in serum is a commonly used biomarker for assessing tumor burden and monitoring treatment response, with elevated levels associated with tumor malignancy (13). Furthermore, emerging biomarkers, such as circulating tumor cells (CTC) (14), circulating tumor DNA (15), circulating microRNAs (16), and NETest (17), present new opportunities for the diagnosis and prognostic evaluation of GI-nets.

However, there are still challenges in the prognostic assessment of GI-net patients. Although the tumor-node-metastasis (TNM) staging system, proposed by Pierre Denoix in 1953, is still the gold standard for oncological prognosis (18). However, it has significant limitations, such as low accuracy and the omission of crucial variables that impact tumor prognosis and treatment options, leading to inadequate predictions of individual survival risk. The varied behavior of NETs makes it difficult to design a practical staging system to provide accurate prognostic information (19). Therefore, it is crucial to establish personalized predictive models for GI-net patients. In recent years, nomograms have been widely used for tumor prognosis (20–22). One major advantage of nomograms is that they can estimate individual risk on the basis of specific patient and disease characteristics. This ability helps doctors create personalized treatment plans, which improves treatment effectiveness and increases patient survival rates.

However, because GI-nets are rare, few studies have focused on them. Currently, there are no individual predictive models to predict the prognosis of patients with GI-nets. This study aimed to develop two nomograms and online risk calculators to predict the OS and CSS of GI-net patients. We used a large dataset from the SEER database for model building and validated it with an external validation group from a single center.

## 2 Methods

### 2.1 Data sources

The SEER database is the authoritative source from the National Cancer Institute (NCI) that tracks cancer incidence and survival rates in the population (23). It has been available since 1973 and is updated every year. Data for related patients diagnosed with GI-nets between 2010–2015 were extracted from the SEER 17 registry database by SEER*Stat 8.4.4 software. Referring to previous research (24–26), they were assigned randomly to the training group or the internal-validation group at a 7:3 ratio. This ratio balance optimizes both model training capacity and validation credibility. 86 GI-net patients treated at the Fourth Hospital of Hebei Medical University from 2010 to 2020 served as the external-validation group. The training group was used to select variables and build the model, whereas the validation group was used to validate the model.

The inclusion criteria were as follows: (1) International Classification of Diseases (ICD) codes O–3 morphology: 8240/3 and 8249/3; (2) primary site codes: C16.0–C16.6, C17.0, C17.1–C17.2, C17.3–C17.9, C18.0, C18.1, C18.2–C18.9, C19.9, C20.9, and C25.0–C25.9; (3) staging on the basis of the seventh edition of the AJCC; (4) age at diagnosis > 18 years; and (5) NET as the only confirmed tumor.

**Figure 1:**
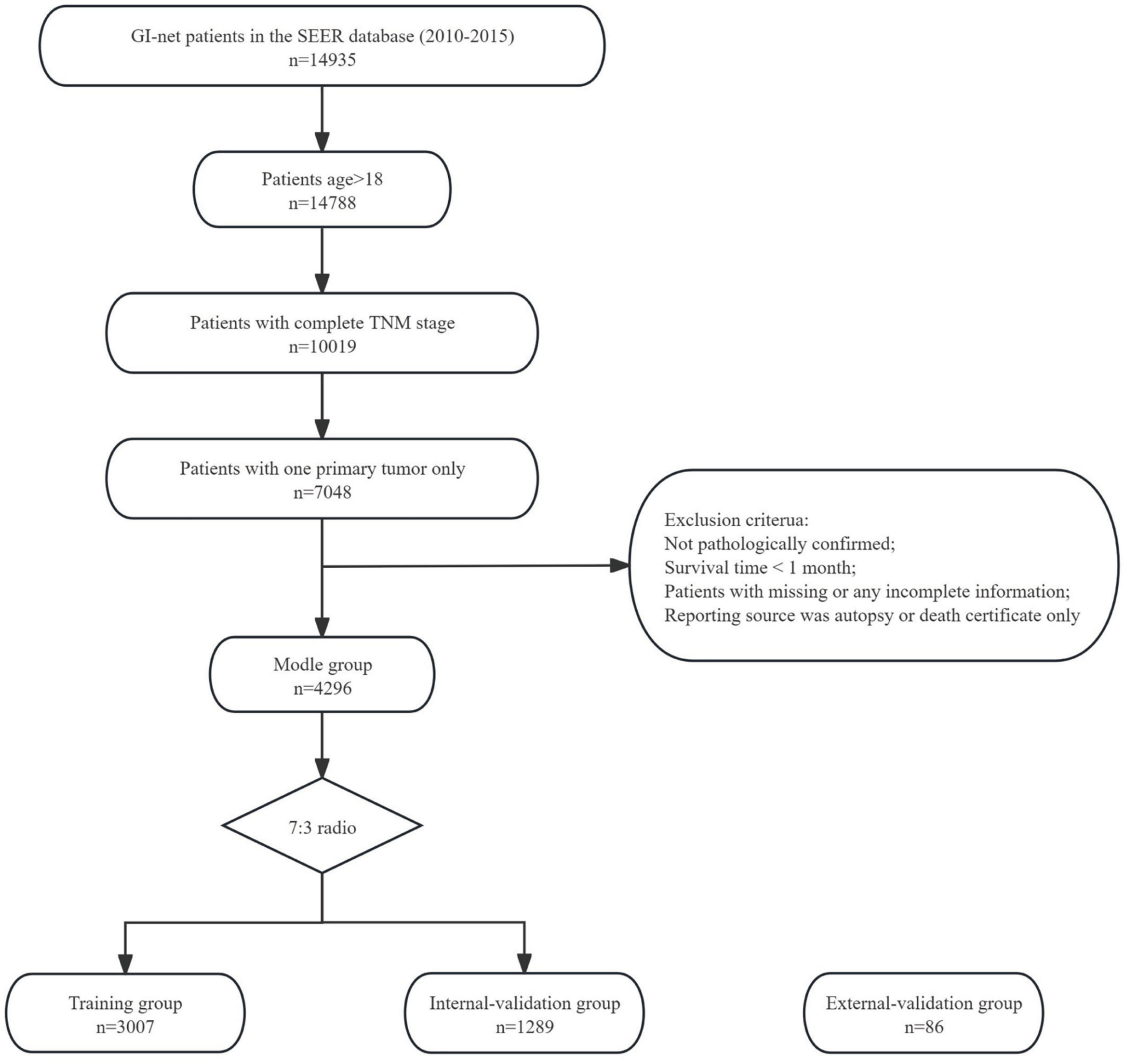
The flowchart of patients inclusion among the SEER database. The flow diagram of selection of patients with GI-net in this study.

The exclusion criteria were as follows: (1) not pathologically confirmed; (2) survival time < 1 month; (3) unknown age, sex, size, T stage, N stage, M stage, treatment methods, etc; and (4) reporting source was autopsy or death certificate only. The specific screening process is shown in **Figure 1**.

### 2.2 Variables

The clinicopathological data of the patients included patient information (age and sex), tumor data (site, size, grade, T, N, and M), treatment methods (surgery, radiotherapy, and chemotherapy), and outcome variables (survival status and survival time (months)). X-tile software was used to evaluate the optimal cutoff values for age and tumor size. The primary endpoint of our study was OS, with a secondary focus on CSS. OS was defined as the interval from the date of initial diagnosis to the date of last follow-up or death from any cause. CSS was defined as the interval from the date of initial diagnosis to the date of last follow-up or death specifically due to neuroendocrine causes.

### 2.3 Ethical approval

This study was approved by the Ethics Committee of the Fourth Hospital of Hebei Medical University (Approval No: 2024KS116), and obtained waiver for informed consent to participate. Our research was conducted in accordance with the Declaration of Helsinki.

### 2.4 Statistical analysis

The categorical variables are presented as percentages, and the continuous variables are presented as the means ± standard deviations (SDs). Univariate Cox regression was used to calculate the hazard ratio (HR) and 95% confidence interval (CI) for each risk factor. Significant factors identified in the univariate analysis were included in the multivariate Cox regression. This inclusion is based on the Akaike information criterion (AIC). In the multivariate analysis, significant clinical variables served as independent predictive factors. These factors were used to create nomograms that predict 3-year and 5-year OS and CSS. The C-index and time‐dependent receiver operating characteristic (ROC) curve were used to evaluate the predictive ability of the nomogram. The C-index and AUC ≥ 0.7 indicate that the nomogram has good predictive discrimination ability. The calibration curve was used to evaluate the difference between the predicted results and the actual results of the nomogram. The closer the predicted calibration curve is to the standard curve, the greater the predictive ability of the nomogram. Decision curve analysis was used to assess the clinical benefit of the nomogram, quantifying the net benefit at different threshold probabilities. KM curves and the log-rank test were used to analyze differences in OS and CSS among the different groups. The p value was used for all the statistical analyses, with p<0.05 defined as statistically significant.

Variance inflation factor (VIF) values were computed to assess the extent of multicollinearity present among the characteristics (27). VIF of >5 indicates that the characteristics are highly correlated and should be considered for removal. Additionally, Pearson’s correlation coefficients were determined to identify collinearity between the characteristics (28). Specifically, a correlation coefficient of <1 between any two independent characteristics was interpreted as an absence of multicollinearity.

Statistical analysis and plotting were completed using R 4.4.1 (http://www.R-project.org/) and the RStudio environment.

## 3 Results

### 3.1 Baseline patient demographics

A total of 4,296 GI-net patients were selected from the SEER database and assigned randomly to the training group (n=3007) or the internal-validation group (n=1289). Additionally, 86 GI-net patients from our hospital were included in the external-validation group. The baseline demographics and clinicopathologic characteristics are listed in **Table 1**. No significant differences in demographics and clinical characteristics were observed between the training and internal-validation groups (all P > 0.05). In the SEER group (n=4296), the average age of patients is 55.55 ± 14.43 years. There are more female patients than male (53.84% vs 46.15%). The most common site of GI-net is the small intestine (42.76%). A majority of patients are in the early stage (T0-2: 69.9%, N0: 68.92%, M0: 90.29%). More patients underwent surgery (95.68%), while fewer received radiotherapy (0.37%) and chemotherapy (2.58%). In the external-validation group (n=86), the average age of patients is 54.45 ± 11.60 years. The most common site of onset is the stomach (43.02%).

**Table 1:** The baseline clinical characteristics of the GI-net patients in training group, internal-validation group, and external-validation group.

Characteristics	SEER group	Training group	Internal-validation group	External-validation group	P (Training vs. Internal-validation group)
Age (years)	55.55 ± 14.43	55.43 ± 14.20	55.82 ± 14.98	54.45 ± 11.60	0.677
Sex					0.565
Female	2313 (53.84)	1635 (54.37)	678 (52.60)	43 (50.00)	
Male	1983 (46.15)	1372 (45.63)	611 (47.40)	43 (50.00)	
Site					0.186
Stomach	370 (8.61)	237 (7.88)	133 (10.32)	37 (43.02)	
Small intestine	1837 (42.76)	1295 (43.07)	542 (42.05)	10 (11.63)	
Cecum	179 (4.17)	123 (4.09)	56 (4.34)	1 (1.16)	
Appendix	674 (15.70)	456 (15.16)	218 (16.91)	5 (5.81)	
Colon	93 (2.16)	73 (2.43)	20 (1.55)	2 (2.33)	
Rectum	1143 (26.60)	823 (27.37)	320 (24.83)	31 (36.05)	
Size (mm)	13.68 ± 13.20	13.67 ± 13.35	13.71 ± 12.58	16.40 ± 19.60	0.998
Grade					0.351
I	3507 (81.63)	2451 (81.51)	1056 (81.92)	49 (56.98)	
II	737 (17.16)	526 (17.49)	211 (16.37)	32 (37.21)	
III-IV	52 (1.21)	30 (0.10)	22 (1.71)	5 (5.81)	
T					0.469
0-2	3003 (69.90)	2085 (69.34)	918 (71.22)	65 (75.58)	
3-4	1293 (30.10)	922 (30.67)	371 (28.78)	21 (24.42)	
N					0.985
0	918 (68.92)	2075 (69.01)	886 (68.74)	64 (74.41)	
1	371 (31.08)	932 (30.99)	403 (31.26)	22 (25.58)	
M					1
0	3879 (90.29)	2715 (90.29)	1164 (90.30)	73 (84.88)	
1	417 (9.71)	292 (9.7)	125 (9.70)	13 (15.12)	
Surgery					0.989
No	187 (4.35)	130 (4.32)	57 (4.42)	12 (13.95)	
Yes	4109 (95.68)	2877 (95.68)	1232 (95.58)	74 (86.05)	
Radiotherapy					0.807
No	4280 (99.63)	2997 (99.67)	1283 (99.53)	1283 (99.53)	
Yes	16 (0.37)	10 (0.33)	6 (0.47)	6 (0.47)	
Chemotherapy					0.989
No	4185 (97.44)	2930 (97.43)	1255 (97.36)	77 (89.53)	
Yes	111 (2.58)	77 (2.56)	34 (2.64)	9 (10.47)	

**Figure 2:**
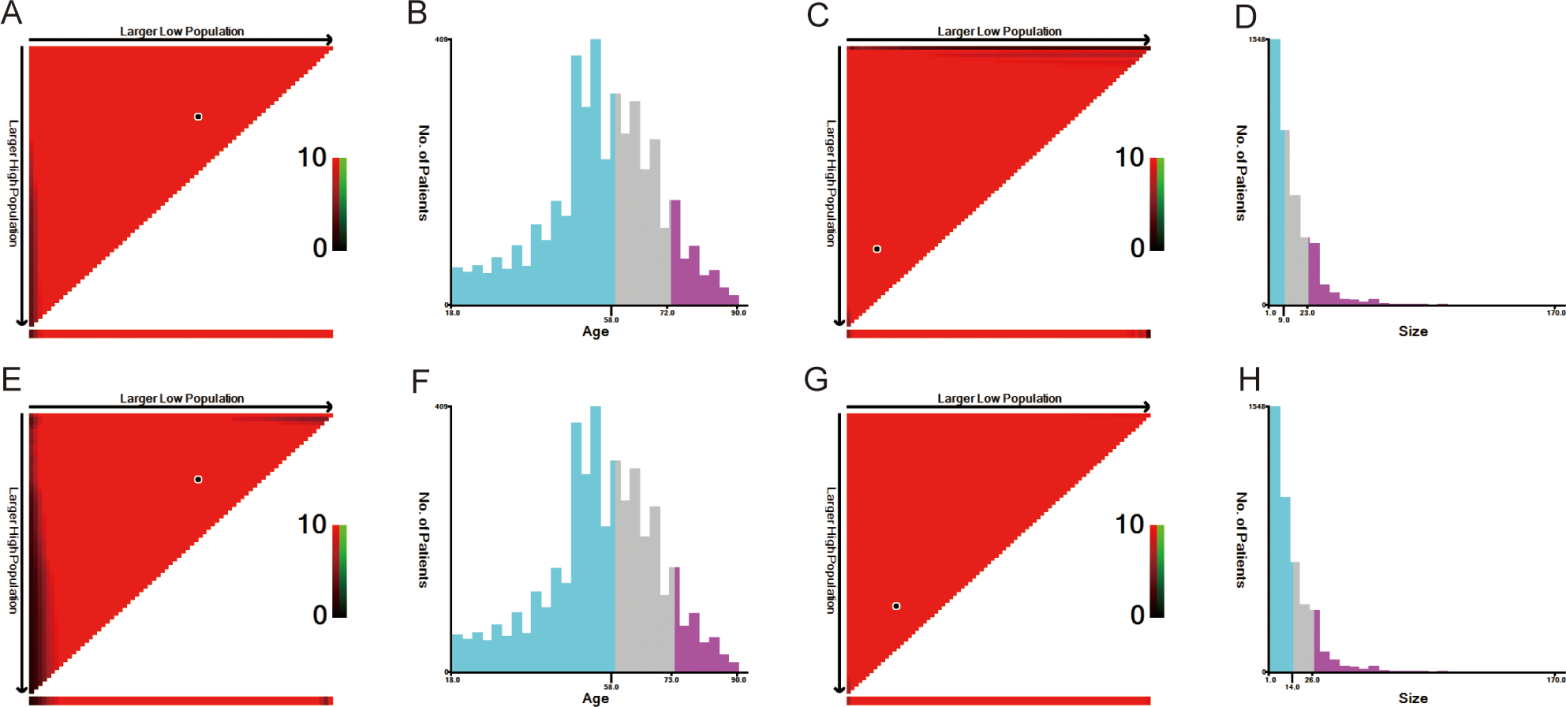
The optimal cut-off value of age and size calculated by X-tile. The age and size data were presented in a triangular grid pattern. Each pixel highlight represented the log-rank teat value [**(A)** age of OS, **(C)** size of OS, **(E)** age of CSS, **(G)** size of CSS], the distribution of the number of patients is shown in the histogram **(B)** age of OS, [**(D)** size of OS, **(F)** age of CSS, **(H)** size of CSS]. OS, overall survival; CSS, cancer-specific survival.

The optimal cutoff values for patient age and tumor size were calculated using X-tile software version 3.6.1 (https://x-tile.software.informer.com/) (**Figure 2**). For OS, the optimal cutoff values for age are 58 and 72 years, and for tumor size are 9 and 23 mm. For CSS, the optimal cutoff values for age are 58 and 73 years, and for tumor size are 14 and 26 mm.

Nomogram variable screening a total of 11 clinical variables were included. According to the univariate Cox regression analysis, only sex was not associated with OS (P=0.08), and CSS was similarly unaffected (P=0.648). The remaining variables were included in the multivariate Cox regression analysis. The multivariate Cox regression analysis revealed that age, site, size, grade, M stage, surgery, and chemotherapy were independent prognostic factors for OS (**Table 2**). For CSS (**Table 3**), age, size, grade, M stage, surgery, and chemotherapy were found to be independent prognostic factors.

**Table 2:** Univariate and multivariate analyses of OS for GI-net patients.

Characteristics	Univariate Cox regression analysis			Multivariate Cox regression analysis		
	HR	95%CI	P	HR	95%CI	P
Age (years)						
≤58	Reference			Reference		
59-72	3.004	2.394-3.768	<0.001	2.521	1.997-3.182	<0.001
≥73	9.482	7.448-11.934	<0.001	7.896	6.141-10.154	<0.001
Sex						
Female	Reference					
Male	1.172	0.981-1.400	0.080			
Site						
Stomach	Reference			Reference		
Small intestine	1.079	0.802-1.451	0.616	0.816	0.585-1.139	0.232
Cecum	1.168	0.747-1.825	0.495	0.853	0.521-1.398	0.529
Appendix	0.207	0.123-0.347	<0.001	0.438	0.257-0.746	0.002
Colon	0.669	0.349-1.283	0.226	0.896	0.463-1.733	0.744
Rectum	0.357	0.248-0.513	<0.001	0.565	0.388-0.824	0.003
Size (mm)						
≤9	Reference			Reference		
10-23	1.921	1.547-2.384	<0.001	1.193	0.915-1.555	0.192
≥24	3.235	2.572-4.070	<0.001	1.645	1.192-2.272	0.002
Grade						
I	Reference			Reference		
II	1.453	1.173-1.799	0.001	1.195	0.960-1.489	0.111
III-IV	6.258	3.896-10.052	<0.001	3.552	2.078-6.071	<0.001
T						
0-2	Reference			Reference		
3-4	2.452	2.053-2.930	<0.001	1.106	0.846-1.446	0.462
N						
0	Reference			Reference		
1	1.784	1.491-2.134	<0.001	0.851	0.671-1.079	0.183
M						
0	Reference			Reference		
1	3.992	3.261-4.886	<0.001	2.424	1.904-3.087	<0.001
Surgery						
No	Reference			Reference		
Yes	0.411	0.299-0.564	<0.001	0.414	0.295-0.580	<0.001
Radiotherapy						
No	Reference			Reference		
Yes	5.825	2.603-13.037	<0.001	1.004	0.402-2.504	0.994
Chemotherapy						
No	Reference			Reference		
Yes	3.791	2.674-5.374	<0.001	1.980	1.340-2.928	0.001

**Table 3:** Univariate and multivariate analyses of CSS for GI-net patients.

Characteristics	Univariate Cox regression analysis			Multivariate Cox regression analysis		
	HR	95%CI	P	HR	95%CI	P
Age (years)						
≤58	Reference			Reference		
59-73	3.137	2.222-4.430	<0.001	2.763	1.924-3.968	<0.001
≥74	6.191	4.106-9.336	<0.001	6.077	3.908-9.450	<0.001
Sex						
Female	Reference					
Male	1.070	0.800-1.430	0.648			
Site						
Stomach	Reference			Reference		
Small intestine	2.423	1.231-4.769	0.010	0.704	0.320-1.513	0.369
Cecum	4.483	2.053-9.789	<0.001	0.861	0.361-2.052	0.735
Appendix	0.175	0.047-0.648	0.009	0.344	0.091-1.299	0.115
Colon	1.727	0.579-5.155	0.327	1.295	0.414-4.052	0.657
Rectum	0.716	0.333-1.540	0.392	0.965	0.430-2.161	0.930
Size (mm)						
≤14	Reference			Reference		
15-26	6.085	3.994-9.272	<0.001	2.456	1.445-4.175	0.001
≥27	15.154	10.009-22.943	<0.001	4.173	2.390-7.284	<0.001
Grade						
I	Reference			Reference		
II	2.193	1.583-3.039	<0.001	1.481	1.053-2.083	0.024
III-IV	17.216	10.189-29.089	<0.001	4.204	2.215-7.982	<0.001
T						
0-2	Reference			Reference		
3-4	7.631	5.422-10.740	<0.001	1.1507	0.919-2.472	0.104
N						
0	Reference			Reference		
1	4.636	3.405-6.313	<0.001	1.330	0.897-1.972	0.156
M						
0	Reference			Reference		
1	13.035	9.741-17.443	<0.001	4.639	3.279-6.563	<0.001
Surgery						
No	Reference			Reference		
Yes	0.408	0.244-0.682	0.001	0.313	0.175-0.558	<0.001
Radiotherapy						
No	Reference			Reference		
Yes	15.344	6.792-34.662	<0.001	0.693	0.258-1.860	0.467
Chemotherapy						
No	Reference			Reference		
Yes	10.552	7.195-15.475	<0.001	2.335	1.490-3.658	<0.001

All the Pearson’s correlation coefficients between pairs of characteristics were < 1 and the VIF values were <5, indicating no collinearity among the independent characteristics (**Supplementary Figure 1**).

### 3.2 Nomogram construction and validation

**Figure 3:**
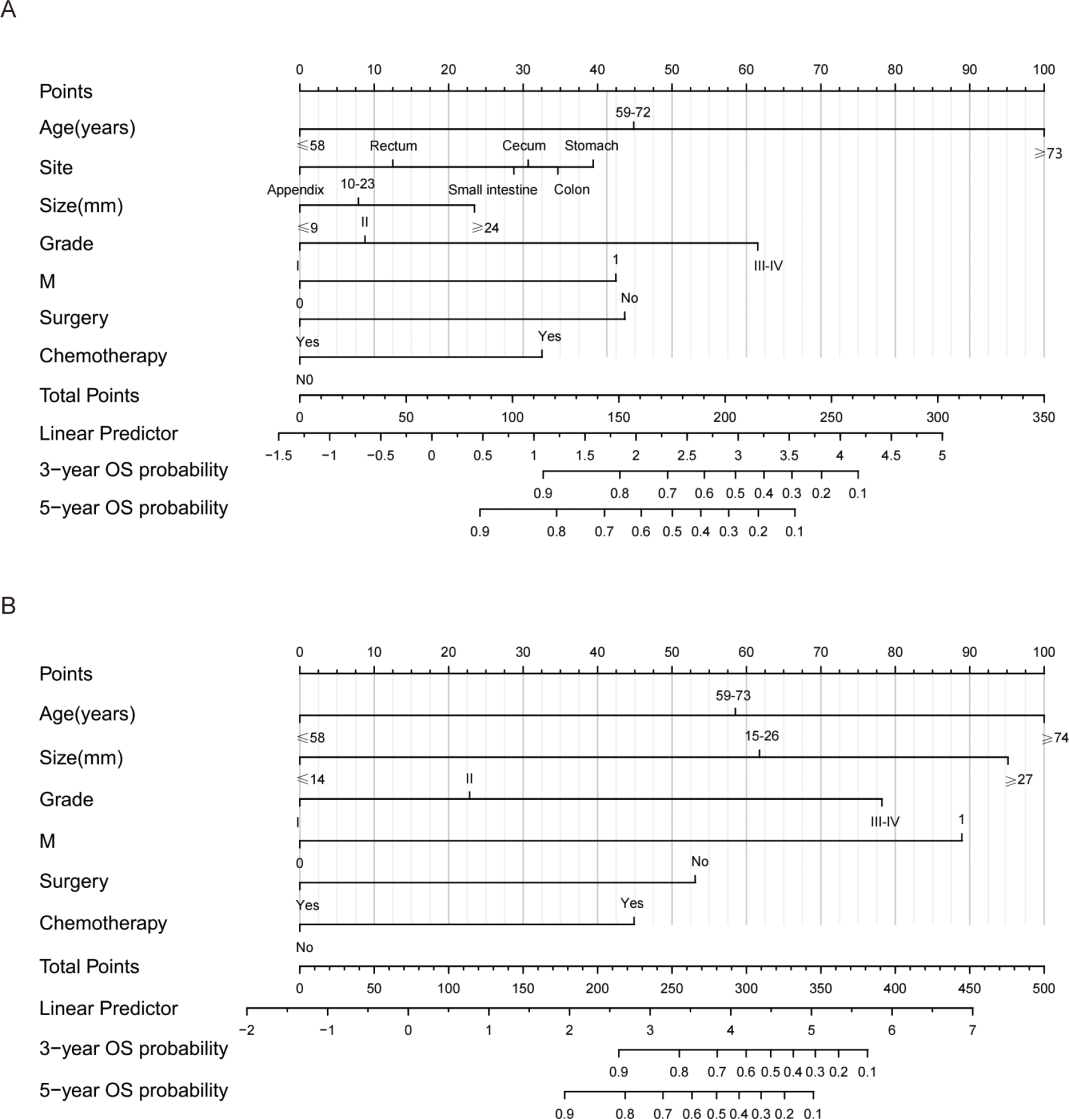
Nomograms for predicting 3- and 5-year OS and CSS of GI-net patients. **(A)** nomogram to predict 3-, and 5-year OS for GI-net patients; **(B)** nomogram to predict 3-, and 5-year CSS for GI-net patients. OS, overall survival; CSS, cancer-specific survival.

Nomograms were constructed to predict the 3-year and 5-year OS and CSS rates using the selected clinical variables (**Figure 3**). Each variable’s value corresponds to a specific individual score. By summing all the individual scores, we can determine the total score. The point where this total score’s vertical line intersects with the predicted probability line indicates the patient’s 3-year and 5-year survival rates. In the nomograms for OS and CSS, age was the most significant variable affecting survival outcomes.

**Figure 4:**
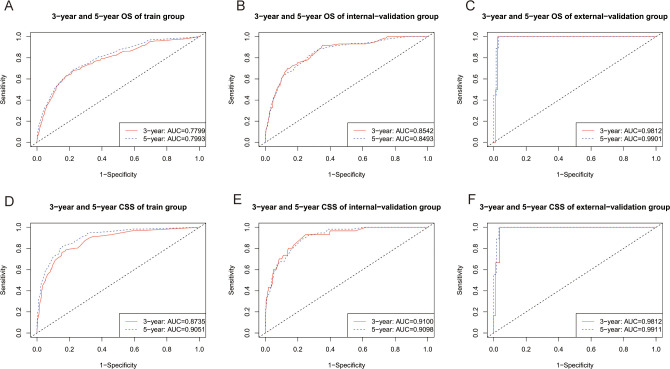
Time-dependent ROC curves of nomograms. **(A)** Time-dependent ROC curves of the OS nomogram showed that the AUCs in the training group were 0.7799,and 0.7993 for predicting 3-year and 5-year OS, respectively. **(B)** Time-dependent ROC curves of the OS nomogram showed that the AUCs in the internal-validation group were 0.8542, and 0.8493 for predicting 3-year and 5-year OS, respectively. **(C)** Time-dependent ROC curves of the OS nomogram showed that the AUCs in the external-validation group were 0.9812, and 0.9901 for predicting 3-year and 5-year OS, respectively. **(D)** Time-dependent ROC curves of the CSS nomogram showed that the AUCs in the training group were 0.8730, and 0.9051 for predicting 3-year and 5-year CSS, respectively. **(E)** Time-dependent ROC curves of the CSS nomogram showed that the AUCs in the internal-validation group were 0.9100 and 0.9098 for predicting 3-year and 5-year CSS, respectively. **(F)** Time-dependent ROC curves of the CSS nomogram showed that the AUCs in the external-validation group were 0.9812 and 0.9911 for predicting 3-year and 5-year CSS, respectively. AUC, area under the time‐dependent receiver operating characteristic curves; OS, overall survival; CSS, cancer-specific survival.

**Figure 5:**
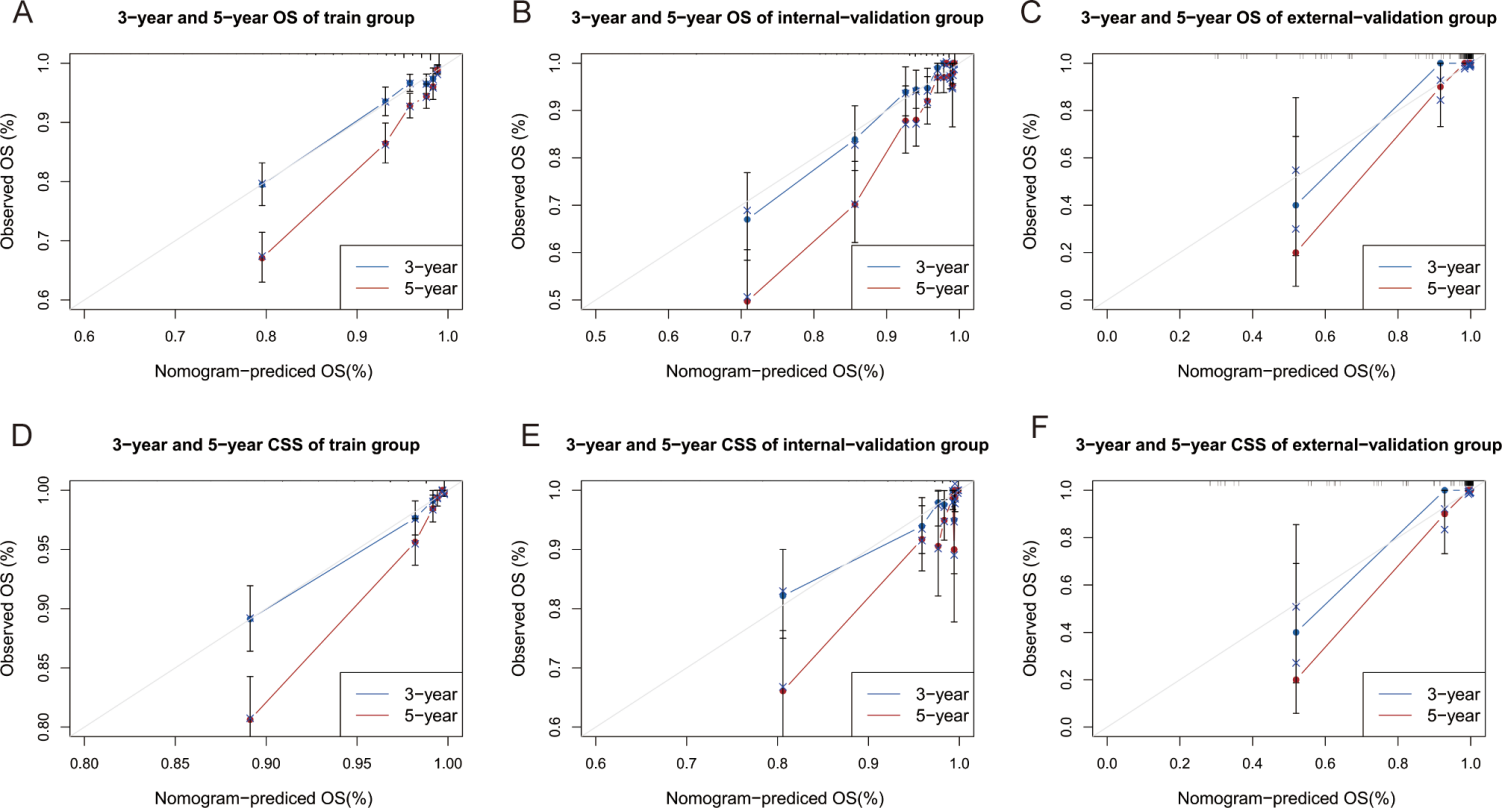
Calibration curves of nomograms. **(A–C)** Calibration curves of 3-year and 5-year OS for GI-net patients in training, internal-validation, and external-validation groups. **(D–F)** Calibration curves of 3-year and 5-year CSS for GI-net patients in training, internal-validation, and external-validation groups. GI-net, gastrointestinal neuroendocrine tumor; OS, overall survival; CSS, cancer-specific survival.

**Figure 6:**
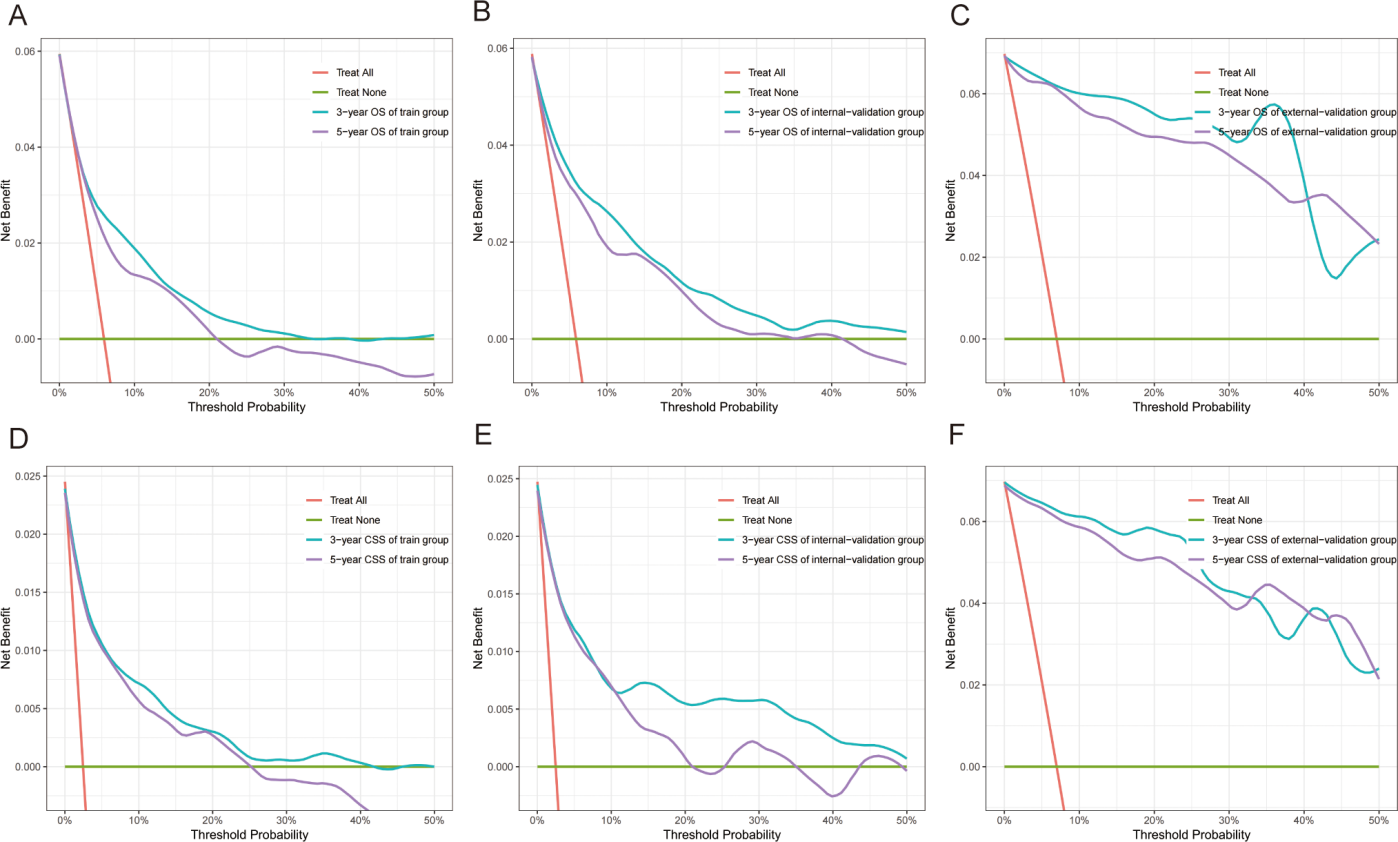
Decision curve analysis (DCA) of nomograms. **(A–C)** DCA of 3-year and 5-year OS for GI-net patients in training, internal-validation, and external-validation groups. **(D–F)** DCA of 3-year and 5-year CSS for GI-net patients in training, internal-validation, and external-validation groups. GI-net, gastrointestinal neuroendocrine tumor; OS, overall survival; CSS, cancer-specific survival.

The C indices for OS were as follows: training group, 0.785 (95% CI: 0.764–0.805); internal-validation group, 0.813 (95% CI: 0.785–0.841); and external-validation group, 0.936 (95% CI: 0.859–1.013). For CSS, the C indices were as follows: training group: 0.888 (95% CI: 0.866–0.911); internal-validation group: 0.893 (95% CI: 0.864–0.922); and external-validation group: 0.930 (95% CI: 0.849–1.011). All the C indices are greater than 0.70. The AUCs of the nomogram for predicting 3- and 5-year OS were 0.780 and 0.799, respectively (**Figure 4A**). The AUCs of the nomogram for predicting 3- and 5-year CSS were 0.874 and 0.905, respectively (**Figure 4D**). The internal-validation group and the external-validation group yielded similar results (≥0.7) (**Figures 4B, C, E, F**). These findings indicate that the two nomograms we established were accurate. The calibration curves (**Figure 5**) revealed high consistency between the predicted OS and CSS probabilities and the actual occurrence probabilities in the three groups. Moreover, DCA (**Figure 6**) revealed that the nomograms had good net clinical benefits. Therefore, our nomograms showed good predictive ability for GI-net patients.

### 3.3 Risk stratification for GI-net patients

**Figure 7:**
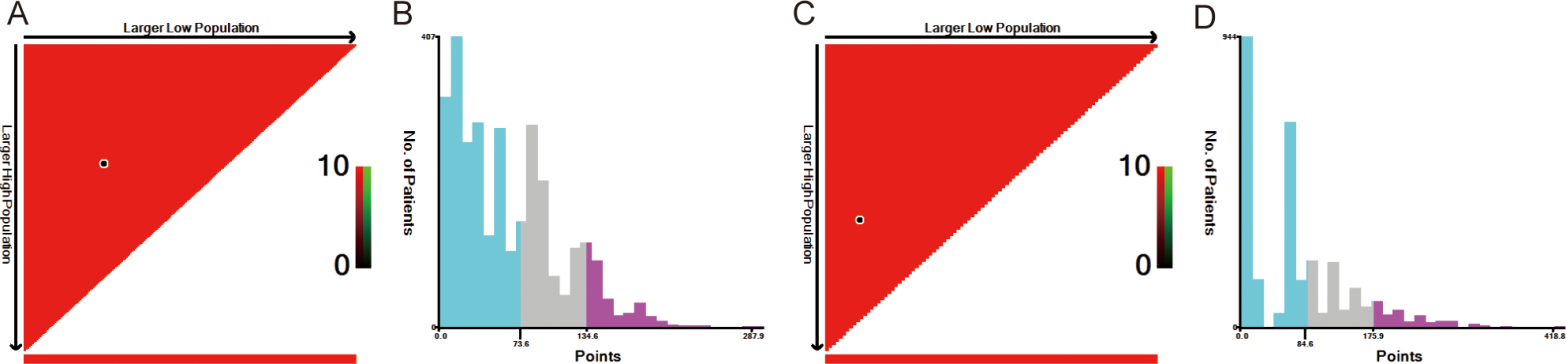
The optimal cut-off value of nomograms score calculated by X-tile. The nomogram score data were presented in a triangular grid pattern. Each pixel highlight represented the log-rank teat value **[(A)** nomogram score of OS, **(C)** nomogram score of CSS], the distribution of the number of patients is shown in the histogram [**(B)** nomogram score of OS, **(D)** nomogram score of CSS]. OS, overall survival; CSS, cancer-specific survival.

**Figure 8:**
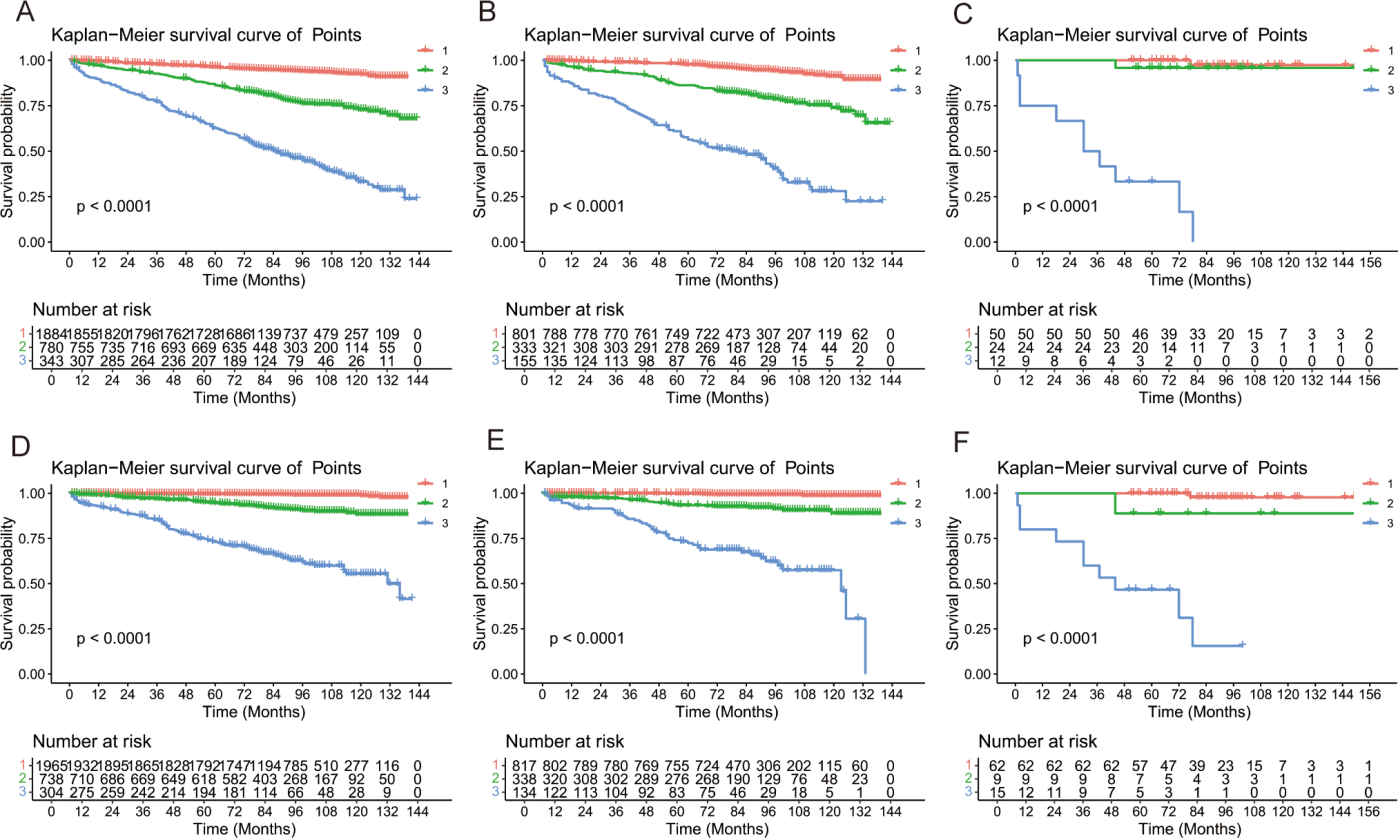
Kaplan–Meier survival analyses for GI-net patients according to the risk stratification. Survival curves showed the OS [**(A)** and CSS **(D)** of the low-risk (1: red),medium-risk (2: green) and high-risk (3: blue) groups in the training group, the OS **(B)** and CSS **(E)** in the internal-validation group, and the OS **(C)**] and CSS **(F)** in the external-validation group. GI-net, gastrointestinal neuroendocrine tumor; OS, overall survival; CSS, cancer-specific survival.

Risk stratification is very important for guiding doctors in patient management. X-tile software was used to determine the optimal cutoff value for risk stratification on the basis of the overall nomogram score of patients (**Figure 7**). Patients were divided into three risk groups: low-risk (OS: points ≤ 73.6; CSS: points ≤ 84.6), medium-risk (OS: 73.6 < points ≤ 134.6; CSS: 84.6 < points ≤ 175.9), and high-risk (OS: points > 134.6; CSS: points > 175.9) groups. KM survival curves and log-rank tests revealed significant differences among the three risk groups (*p* < 0.001) in the training cohort (**Figure 8**). The internal-validation group and the external-validation group yielded similar results. These findings indicate the effectiveness of the nomogram risk stratification system.

### 3.4 Web-based online risk calculator

To facilitate clinical application, we created two online risk calculators based on the shinyapp.io platform (**Supplementary Figure 2**). These calculators allow doctors to quickly understand patients’ survival probabilities by selecting clinical variables. The web-based OS online risk calculator can be accessed at https://liexiantusc.shinyapps.io/DynNomapp_OS/; the web-based CSS online risk calculator can be accessed at https://dynnom-sc.shinyapps.io/DynNomapp_CSS/.

## 4 Discussion

The incidence and mortality rates of GI-nets are increasing due to advancements in examination technologies such as endoscopy and imaging, alongside increased health awareness and understanding of diseases (6, 9, 10). The SEER database is an important cancer epidemiology database created and maintained by the NCI in the United States (29). This database provides extensive epidemiological data on GI-nets. In this study, we explored the clinicopathological characteristics and prognostic factors of GI-net patients using data from the SEER database and our hospital’s single-center data.

Previous studies have confirmed the predictive ability of nomograms for gastric (30), small intestine (31, 32), appendiceal (33), and colorectal (34) NETs. These studies all show that clinically applicable nomograms can accurately predict the prognosis of NET patients. Our research involves GI-nets originating from the stomach, small intestine, appendix, cecum, and colon as study characteristics and identifies them as independent prognostic factors for OS. Unfortunately, they have no significant impact on the CSS.

Compared with the previous nomogram by Wu et al. (35) for gastroenteropancreatic neuroendocrine tumors (GEP-nets), our nomogram has several differences. First, pancreatic neuroendocrine tumors (P-nets) and GI-nets have different origins, with P-nets originating from pancreatic neuroendocrine cells (Langerhans tumor cells) and GI-nets originating from enterochromaffin cells (36). Additionally, their pathogenesis, biological behaviors, and treatment methods differ (37). In terms of molecular mechanisms, GI-nets carry APC gene mutations, while P-nets carry mutations in ATRX, FOXO3, and PTEN genes (38). In terms of biological behavior, the metastasis rate of GI-nets is 2.2% to 5.6%, significantly lower than the 34% for P-nets (39). In terms of treatment, the chemotherapy regimens for there are different, and P-nets chemotherapy is used more frequently and show better efficacy (40). Therefore, it is necessary to establish a separate personalized prediction model for GI-net patients. Moreover, our study endpoints included not only OS but also CSS. We have also created online risk calculators. Finally, our nomograms were developed in the training group and validated in both the internal- and external-validation groups; thus, the results have high credibility. This comprehensive analysis is also an important improvement in our research.

Using data from the SEER database and applying univariate and multivariate Cox regression analyses, we identified age, site, size, grade, M stage, surgery, and chemotherapy as risk factors influencing OS, whereas age, size, grade, M stage, surgery, and chemotherapy were found to affect CSS. The C-index, ROC curve, calibration curve, and DCA curve all indicate that the nomograms exhibited strong predictive performance. Additionally, our two nomograms performed exceptionally well in both the internal- and external-validation groups. Furthermore, patients can be effectively divided into high-, medium-, and low-risk groups on the basis of the risk stratification of the nomogram. Patients defined as high-risk by the nomogram are expected to have a poor prognosis. Therefore, we recommend that these patients receive additional treatment and close follow-up. Moreover, for the first time, we have developed two online risk calculators that can predict the OS and CSS of GI-net patients and provide visible results, aiming to offer more references for personalized treatment.

Consistent with previous studies (41–43), our research revealed that increasing age is associated with poorer prognosis. This may be related to the decline in immune system function associated with aging, making tumors more likely to evade the host’s immune defenses (44, 45). Our analysis revealed that patients with GI-nets diagnosed at the localized stage had better outcomes than those with regional or distant disease. These findings underscore the critical importance of early detection and treatment of NETs. Interestingly, traditional “T” and “N” stages were not included as independent prognostic factors for OS or CSS. This suggests that traditional TNM staging has certain limitations and highlights the necessity of constructing a prognostic model specifically for GI-net patients.

Despite the good performance of the nomograms, there are still several limitations. First, this is a retrospective study based on the SEER database and a single center in our hospital, so selection bias is unavoidable. In addition, the SEER database does not provide genetic data, Ki-67 levels, or radiotherapy or chemotherapy regimens, which prevents us from evaluating the impact of gene mutations, cellular proliferation activity, or treatment regimens on OS and CSS in patients with GI-nets. As a result, the predictive accuracy could be limited for GI-net patients with specific genetic mutations, high proliferation activity, or those undergoing particular treatment regimens. Future multicenter studies are needed to integrate molecular markers and treatment details to further optimize predictive models. Finally, due to the limited size of the external-validation group, additional multicenter prospective validation studies are needed. Despite these limitations, this is a large population-based study that investigated the prognostic factors of GI-net patients and confirmed that the nomograms have good predictive ability.

## 5 Conclusion

We established and validated two new nomograms and online risk calculators to predict 3- and 5-year OS and CSS for GI-net patients. Our nomograms demonstrated satisfactory accuracy and effectively distinguished between three different risk groups of patients. This may assist with precision and personalized treatment for GI-net patients.

## Data Availability

The datasets presented in this study can be found in online repositories. The names of the repository/repositories and accession number(s) can be found below: SEER database, http://seer.cancer.gov/seerstat.
