# Supplementary material for: Dynamic nomogram for predicting the overall survival and cancer-specific survival of patients with gastrointestinal neuroendocrine tumor: a SEER-based retrospective cohort study and external validation
Source: Front Oncol. 2025 Jun 4;15:1594591. doi: 10.3389/fonc.2025.1594591 (PMC12173924; doi:10.3389/fonc.2025.1594591)
Supplement: Supplementary file 1 [file DataSheet1.pdf]

Supplementary Material

1. Supplementary Figures

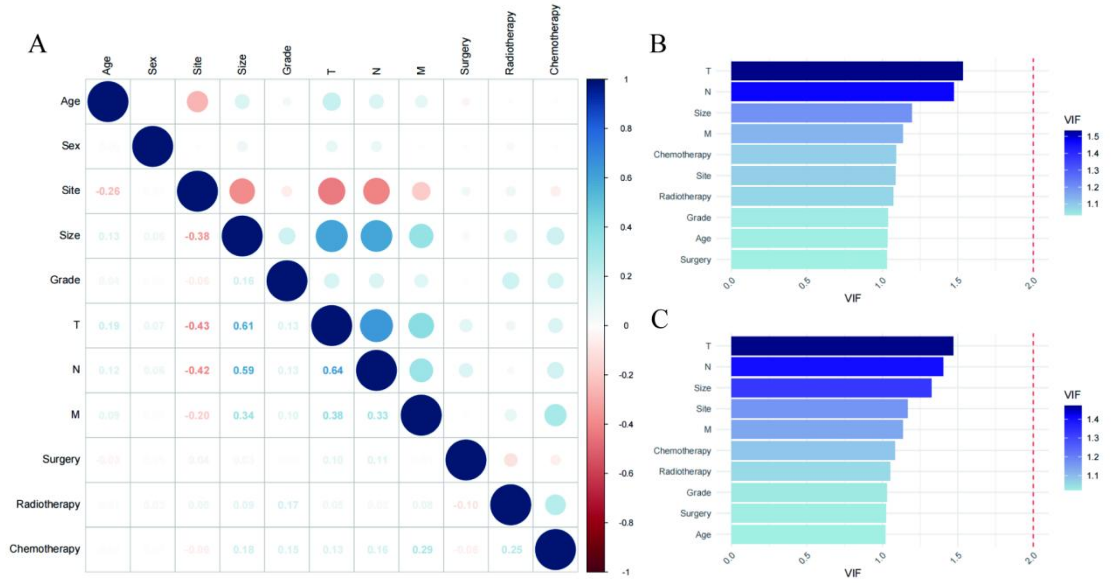

Supplementary Figure 1. Multicollinearity analysis. (**A)** Pearson's correlation coefficients between pairs of characteristics. (**B**) VIF of OS characteristics. (**C**) VIF of CSS characteristics. VIF, variance inflation factor; OS, overall survival; CSS, cancer-specific survival

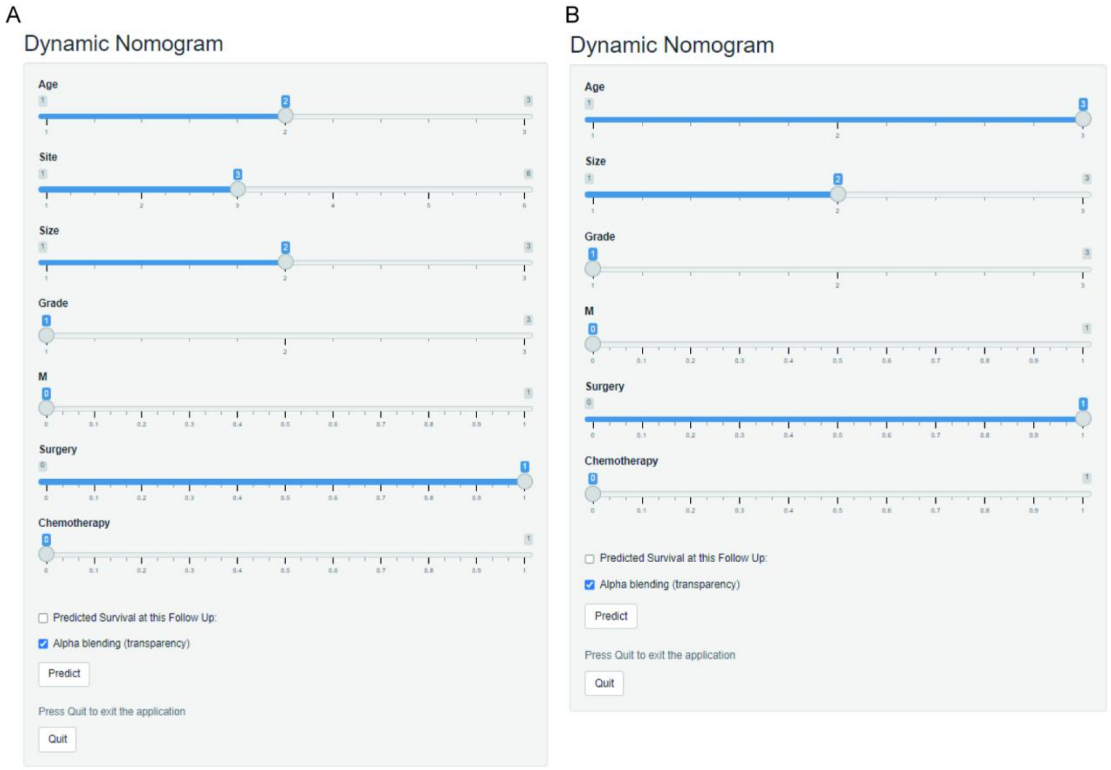

Supplementary Figure 2. Web-based online risk calculators for predicting 3- and 5-year OS and CSS of GI-net patients. (**A**) Web-based online risk calculator to predict 3-, and 5-year OS for GI-net patients; (**B**) Web-based online risk calculator to predict 3-, and 5-year CSS for GI-net patients. OS, overall survival; CSS, cancer-specific survival
